# A spoon full of studies helps the comparison go down: a comparative analysis of Tulving’s spoon test

**DOI:** 10.3389/fpsyg.2014.00893

**Published:** 2014-08-12

**Authors:** Damian Scarf, Christopher Smith, Michael Stuart

**Affiliations:** Department of Psychology, University of OtagoDunedin, New Zealand

**Keywords:** mental time travel, episodic memory, episodic foresight, Tulving’s spoon test, comparative cognition

## Abstract

Mental time travel refers to the ability to cast one’s mind back in time to re-experience a past event and forward in time to pre-experience events that may occur in the future. Tulving (2005), an authority on mental time travel, holds that this ability is unique to humans. Anticipating that comparative psychologists would challenge this claim, Tulving (2005) proposed his spoon test, a test specifically designed to assess whether non-human animals are capable of mental time travel. A number of studies have now employed the spoon test to assess mental time travel in non-human animals. Here, we review the evidence for mental time travel in primates. To provide a benchmark, we also review studies that have employed the spoon test with preschool children. The review demonstrates that if we compare the performance of great apes to that of preschool children, and hold them to the same criteria, the data suggest mental travel is present but not ubiquitous in great apes.

At its heart, comparative psychology is founded on the principle of continuity. Darwin (1871) articulated this principle perfectly when he stated that any differences between human and non-human animal minds are differences of degree (i.e., quantitative) but not kind (i.e., qualitative). Undoubtedly, the vast and growing comparative literature is consistent with Darwin’s (1871) view, however, several recent reviews have suggested that discontinuities may also exist (Tulving, 2005; Premack, 2007; Suddendorf and Corballis, 2007; Penn et al., 2008). On this point, views range from Penn et al.’s (2008, pp. 110) contention that Darwin (1871) was mistaken and overlooked the “profound functional discontinuity between the human and non-human mind” to a more nuanced view suggesting that, although the principle of mental continuity largely holds true, there are examples of discontinuity in mind between human and non-human animals (Tulving, 2005). In the current review we tackle one of the more widely asserted discontinuities – mental time travel (Suddendorf and Corballis, 1997, 2007; Roberts, 2002; Suddendorf and Busby, 2003; Tulving, 2005). The term mental time travel was coined by Suddendorf and Corballis (1997) and denotes the ability to cast one’s mind not only back in time to re-experience a past event (i.e., episodic memory) but also forward in time (i.e., episodic foresight) to pre-experience events that may occur in the future.

Tulving (1983) initially conceptualized mental time travel purely in terms of the recall of past events (i.e., what) and their spatial (i.e., where) and temporal (i.e., when) context (Tulving, 1983). However, the fact that an individual could remember these aspects of an event without having personally experiencing it, led to a reconceptualization that included autonoetic consciousness or the knowledge that one’s memory of an event is a product of them having personally experienced it (Tulving, 1985). Finally, Tulving (2002) added proscopic chronesthesia (i.e., foresight), suggesting the processes used to re-experience past events could also be utilized to pre-experience future events. Fittingly, recent functional magnetic imaging research supports Tulving’s view, demonstrating that the same brain areas are active when a person is asked to reflect on a past event and simulate a future event (Addis et al., 2007; Buckner and Carroll, 2007; Schacter et al., 2012).

While Tulving’s (1983) conceptualization of mental time travel has changed over time, his view that it is a uniquely human ability has remained unchanged. For example, Tulving opened his seminal book, *Elements of Episodic Memory*, by stating “Remembering past events is a universally familiar experience. It is also a uniquely human one” (Tulving, 1983, pp. 1) and more than 20 years later he restated this thesis, “Human beings possess a form of memory (episodic memory) and a form of consciousness (autonoetic consciousness, or “autonoesis”) that no other animals do. Thus, the thesis is that these two aspects of the mind are unique in humans, in the sense that the mental capacities that define them do not exist in quite the same full-fledged form in other species. They do not exist in insects, in birds, in mice or rats, in cats or dogs, and not even in gorillas and chimps” (Tulving, 2005, pp. 6). In addition to restating his thesis, Tulving (2005) also proposed the spoon test, a paradigm that would allow one to test it his claim that mental time travel is uniquely human. At present, we believe the spoon test is the best test of mental time travel that can be used with both young children and non-human animals and, consequently, it forms the backbone of this comparative review.

The spoon test builds on earlier proposals by Köhler (1922) and Suddendorf (1994), both of which highlighted the potential significance of demonstrating that a non-human animal could prepare in the present for a temporally distant event. To describe the spoon test, Tulving refers to an Estonian children’s story in which *“… a young girl dreams about going to a friend’s birthday party where the guests are served delicious chocolate pudding, her favorite. Alas, all she can do is to watch other children eat it, because everybody has to have her own spoon, and she did not bring one. So the next evening, determined not to have the same disappointing experience again, she goes to bed clutching a spoon in her hand”* (Tulving, 2005, pp. 44). According to Tulving (2005) the young girl’s behavior demonstrates that she is able to reflect on her experience of being at the party without a spoon (i.e., episodic memory) and, by placing the spoon underneath her pillow, shows that she can entertain the possibility she may again attend the party in her dreams (i.e., episodic foresight). Tulving (2005, pp. 44) suggests that if a non-human animal were to pass an analogous version of the spoon test it would “force the rejection” of his hypothesis that mental time travel is uniquely human.

## DEVELOPMENTAL STUDIES

Developmental studies of mental time travel provide an important comparison when assessing mental time travel in non-human animals. Those working with non-human animals have been set an ever growing number of criteria that their spoon tests must adhere to (Tulving, 2005; Suddendorf and Corballis, 2007, 2010; Suddendorf et al., 2009), however, one could argue that these criteria are only relevant in so far as they have been upheld in developmental studies that have concluded young children are capable of mental time travel (Suddendorf and Busby, 2005; Russell et al., 2010; Suddendorf et al., 2011; Scarf et al., 2013; Atance and Sommerville, 2014; Payne et al., 2014). To this end, we will first review the small number of developmental studies that have employed the spoon test to assess mental time travel in young children.

Suddendorf et al. (2011) presented 3- and 4-year-old children with a novel problem, in which a specially shaped key could be used to open a locked box. In the first room, children were shown a box with a triangle or cross shaped keyhole and the experimenter demonstrated how a key that matched the shape of the keyhole could be used to open it, revealing several stickers. Children were then given the opportunity to perform the task twice themselves, obtaining a sticker each time. Children were then distracted and the key they had previously used to open the box was replaced by a broken key. After demonstrating the broken key could not be used to open the box, the experimenter ushered the child into another room where they played games for 15 min. After 15 min children were presented with four differently shaped keys, one of which matched the key they used to open the box, and were told they could pick one key to take with them back to the first room. While the majority of 4-year-olds (65%) chose the correct key, the performance of the 3-year-olds (29%) was not significantly above chance.

Using a problem similar to that used by Suddendorf et al. (2011) and Scarf et al. (2013) had 3- and 4-year-old children dig up a locked treasure chest in a large outdoor sandbox. After establishing that they did not have a key to open the treasure chest, the experimenter asked children to go back to the lab with them. Children then left the lab and returned after a 24 h delay. When they returned to the lab, children were told they would be going back out to the sandbox and were asked to pick one of three items (a key, windup toy, or bouncy ball) to take with them. While a significant number of 4-year-old children selected the key, the performance of 3-year-old children was no different from chance. To further investigate the impact of the delay on the performance of the 3-year-old children, separate groups of 3-year-old children were tested after a 0, 15, or 30 min delay. The performance of the 3-year-old children decreased in a linear fashion over the 0, 15, and 30 min delays. The impact of the delay on the performance of 3-year-old children (Scarf et al., 2013) suggests that, while 3-year-old children are capable of mental time travel, they are constrained by their ability to retain the original episode. Indeed, Atance and Sommerville (2014) have demonstrated that if memory of the original episode is controlled for, there is no difference between the performance of 3- and 4-year-old children on the spoon test.

One potential limitation of the developmental studies reviewed above is that they did not include a delay between the selection phase and children being given the opportunity to use the item they selected. Thus, the studies only tested children’s foresight for the very next event (Redshaw and Suddendorf, 2013). However, building on Suddendorf et al. (2011) and Redshaw and Suddendorf (2013) recently demonstrated that the performance of 4-year-old children on the spoon test is not impacted by inserting a 5-min delay after the selection phase, suggesting that the mechanism used to plan for the very next event may be the same mechanism used to plan for more distant events. It will be important for future studies to investigate the potential impact of longer delays. In addition, it is an open question as to whether 3-year-old children are also unaffected by the imposition of a delay after the selection phase.

## COMPARATIVE STUDIES

Comparative studies of the spoon test have employed a different procedure to that used in the majority of the developmental studies reviewed above (cf. Redshaw and Suddendorf, 2013)^1^. Specifically, rather than insert the delay between the original episode and the selection phase, comparative studies have inserted the delay after the selection phase (cf. Beran et al., 2012). This difference somewhat complicates the comparison between studies, because in all the comparative studies there are examples of a subject selecting the correct tool, but failing to transport it to the testing room following the delay. However, given that a subject may simply select the correct tool due to the fact it has previously used it to obtain food, for the comparative studies we will define a successful trial as a subject selecting the correct tool, taking it with them to the delay room, and transporting it to the testing room^2^.

The first comparative study was conducted by Mulcahy and Call (2006), who taught bonobos and orangutans to use a tool to retrieve a reward from an apparatus. After learning this, the apes were presented with several tools (two suitable and six unsuitable) in the testing room but with access to the baited apparatus blocked. After 5 min, in which they were allowed to choose freely between the items, the apes were ushered out of the testing room and any tools that remained in the test room were removed. After a 1 h delay, the apes were allowed back into the testing room with the baited apparatus now accessible. Across 16 trials, subject’s performance ranged from 13–94% (**Table 1**). However, while the performance of subjects demonstrates they are capable of returning to the test room with the tool after a significant delay, any conclusions drawn from this study are somewhat tempered by the fact the animals made their selections with the apparatus in view, raising the possibility that tool selection was cued.

**Table 1:** The trial-by-trial performance of several primate species.

Species	Individual	Delay	1	2	3	4	5	6	7	8	9	10	11	12	13	14	15	16	17
*Macaca fascicularis*	Anastasia^1^	5 min	0	0	0	0	0	0	0	0	0	0	0	0	0	0	0	0	0
	Ekzekwo^1^	5 min	0	0	0	0	0	0	0	0	0	0	0	0	0	0	0	0	0
	Era^1^	5 min	0	0	0	0	0	0	0	0	0	0	0	0	0	0	0	0	0
	Icetea^1^	5 min	0	0	0	0	0	0	0	0	0	0	0	0	0	0	0	0	0
	Ophelia^1^	5 min	0	0	0	0	0	0	0	0	0	0	0	0	0	0	0	0	0
	Zargasso^1^	5 min	0	0	0	0	0	0	0	0	0	0	0	0	0	0	0	0	0
*Pan troglodytes*	Phil^2^	60 min	0	0	0	0	0	0	0	0	0	0	0	0	0	0	0	0	0
	Ton^2^	60 min	0	0	0	0	0	0	0	0	0	0	0	0	0	0	0	0	0
	Femma^2^	60 min	0	0	0	0	0	0	0	0	0	0	0	0	0	0	0	0	0
	Peggy^2^	60 min	0	0	0	0	0	0	0	0	0	0	0	0	0	0	0	0	0
	Thomas^2^	60 min	0	1	0	0	0	0	1	1	0	0	0	0	0	0	1	1	0
	Kenny^2^	60 min	0	0	0	0	0	0	0	0	0	0	0	0	0	0	0	0	0
	Zorro^2^	60 min	0	0	0	0	0	0	0	0	0	0	0	0	0	0	0	0	0
	Juus^2^	60 min	0	0	0	0	0	1	1	1	0	0	0	0	0	1	0	1	0
	Iris^2^	60 min	0	0	0	0	0	0	0	0	0	0	0	0	0	0	0	0	0
	Willy^2^	60 min	0	0	0	0	0	1	1	1	0	0	0	0	1	0	1	1	1
	Linda^3^	70 min	1	1	0	1	1	1	1	1	0	0	1	1	1	1	-	-	-
	Maria^3^	70 min	1	1	1	1	0	1	1	1	1	0	1	1	1	1	-	-	-
*Pan paniscus*	Kuno^4^	60 min	0	0	0	0	0	0	1	1	0	1	0	0	1	1	1	1	-
	Joey^4^	60 min	1	0	0	0	0	0	0	0	0	0	0	0	1	0	0	0	-
	Limbuko^4^	60 min	0	0	0	0	0	0	1	0	0	1	1	0	1	0	0	1	-
*Pongo pygmaeus*	Walter^4^	60 min	0	0	0	1	1	1	1	1	0	0	0	0	0	1	0	0	-
	Toba^4^	60 min	1	0	1	0	0	1	0	0	0	1	1	1	1	0	0	0	-
	Dokana^4^	60 min	1	0	1	1	1	1	1	1	1	1	1	1	1	1	1	1	-
*Pongo abelii*	Naong^3^	70 min	0	1	1	1	1	1	1	0	1	1	0	1	1	1	-	-	-

*^1^Dekleva et al. (2012), Experiment 1a; ^2^Dufour and Sterck (2008), Experiment 5; ^3^Osvath and Osvath (2008), Experiment 1; ^4^Mulcahy and Call (2006), Experiment 1. 0 indicates an incorrect trial, 1 indicates a correct trial, and – indicates no trial.*

Independently of Mulcahy and Call (2006) and Osvath and Osvath (2008) trained two chimpanzees and one orangutan to use a plastic hose to suck fruit soup from an apparatus. Once subjects had learned this, they were called individually into a selection room and given the opportunity to select one of four items, one of which was the plastic hose. After selecting an item, subjects were ushered out of the selection room and back into their enclosure. Critically, from the selection room, subjects were not able to see the apparatus or the room that it was housed in. After a 1-h delay, subjects were allowed access to the testing room. Across 14 trials, subject’s performance ranged from 79–86% (**Table 1**). In a second experiment, Osvath and Osvath (2008) went one step further by presenting a grape as one of the items subjects could choose. Impressively, the performance of all three subjects was comparable to that of the first experiment, suggesting that they were able to forgo the immediate reward in order to acquire a better reward in the future.

In contrast to Mulcahy and Call (2006) and Osvath and Osvath (2008), Dufour and Sterck (2008) found little evidence chimpanzees were capable of passing the spoon test. Dufour and Sterck (2008) trained 10 chimpanzees to use a hook to obtain a bottle of juice placed outside of their individual feeding compartment. During testing, four categories of objects (hooks, straws, branches, and sticks) were made available in a compartment that all subjects could access. After 10 min, the items that remained in the compartment were removed. Testing occurred 1 h later, with subjects invited to enter their feeding compartment. Surprisingly, across 17 trials, only three subjects performed at least one trial correctly (**Table 1**).

It is important to note that Dufour and Sterck’s (2008) study, although including a standard version of the spoon test, focused largely on an exchange version of the spoon test in which subjects exchanged a token with a human, rather than used a tool, to acquire food. Consistent with the performance of their chimpanzees on the standard spoon test, the chimpanzees also performed poorly on the exchange version of the task (Dufour and Sterck, 2008). Two recent studies, however, have demonstrated that chimpanzees (Osvath and Persson, 2013) and orangutans and bonobos (Bourjade et al., 2014) can also successfully pass this task.

As a whole, the comparative studies clearly show that there is marked individual variation in the performance of several great ape species on the spoon test. The high performance of some individuals, however, suggests that, although not ubiquitous, some great apes appear capable of mental time travel (**Table 2**).

**Table 2:** The overall performance of several primate species compared to chance.

Species	Individual	%	Selection	Transport	Overall	*p*-value	Rank	Alpha	Result
*Macaca fascicularis*	Anastasia^1^	0	0.33	0.5	0.17	0.045	12	0.0036	*ns*
	Ekzekwo^1^	0	0.33	0.5	0.17	0.045	13	0.0038	*ns*
	Era^1^	0	0.33	0.5	0.17	0.045	14	0.0042	*ns*
	Icetea^1^	0	0.33	0.5	0.17	0.045	15	0.0045	*ns*
	Ophelia^1^	0	0.33	0.5	0.17	0.045	16	0.0050	*ns*
	Zargasso^1^	0	0.33	0.5	0.17	0.045	17	0.0056	*ns*
*Pan troglodytes*	Phil^2^	0	0.25	0.5	0.13	0.103	18	0.0063	*ns*
	Ton^2^	0	0.25	0.5	0.13	0.103	19	0.0071	*ns*
	Femma^2^	0	0.25	0.5	0.13	0.103	20	0.0083	*ns*
	Peggy^2^	0	0.25	0.5	0.13	0.103	21	0.0100	*ns*
	Thomas^2^	29	0.25	0.5	0.13	0.038	10	0.0031	*ns*
	Kenny^2^	0	0.25	0.5	0.13	0.103	22	0.0125	*ns*
	Zorro^2^	0	0.25	0.5	0.13	0.103	23	0.0167	*ns*
	Juus^2^	29	0.25	0.5	0.13	0.038	11	0.0033	*ns*
	Iris^2^	0	0.25	0.5	0.13	0.103	24	0.0250	*ns*
	Willy^2^	41	0.25	0.5	0.13	0.002	7	0.0026	*s*
	Linda^3^	79	0.25	0.5	0.13	0.000	3	0.0022	*s*
	Maria^3^	86	0.25	0.5	0.13	0.000	2	0.0021	*s*
*Pan paniscus*	Kuno^4^	44	0.25	0.5	0.13	0.002	5	0.0024	*s*
	Joey^4^	13	0.25	0.5	0.13	0.289	25	0.0500	*ns*
	Limbuko^4^	31	0.25	0.5	0.13	0.031	9	0.0029	*ns*
*Pongo pygmaeus*	Walter^4^	38	0.25	0.5	0.13	0.008	8	0.0028	*ns*
	Toba^4^	44	0.25	0.5	0.13	0.002	6	0.0025	*s*
	Dokana^4^	94	0.25	0.5	0.13	0.000	1	0.0020	*s*
*Pongo abelii*	Naong^3^	79	0.25	0.5	0.13	0.000	4	0.0023	*s*

*^1^Dekleva et al. (2012), Experiment 1a; ^2^Dufour and Sterck (2008), Experiment 5; ^3^Osvath and Osvath (2008), Experiment 1; ^4^Mulcahy and Call (2006), Experiment 1. Chance (Overall) was calculated by multiplying the chance of the subject selecting the correct tool (Selection), based on the number of target and non-target tools made available, by the chance of the subject returning with the correct tool following the delay (Transport). The chance of a subject transporting the tool, given all studies coded it as a binary outcome, was set at 0.5. The performance of each subject was assessed using a binomial probability test. Given the large number of tests conducted, a modified Bonferroni correction was employed (Holm, 1979). Holm’s (1979) adjustment uses the rank of each *p*-value (Rank) to calculate the adjusted alpha (Alpha).*

## ONE TRIAL TO RULE THEM ALL

Our conclusion that some great apes appear capable of mental time travel is based on using the same criteria we (Scarf et al., 2013) and others (Suddendorf et al., 2011) have used with preschool children. One issue that must be addressed, however, is the number of test trials the developmental and comparative studies employ. While developmental studies have universally tested children on only 1 trial, the comparative studies have tested great apes on between 14 and 17 trials of the same problem. Suddendorf and Corballis (2010) have argued that mental time travel can only be inferred if single trials are used due to multiple trials (a) potentially resulting in associative learning and, (b) raising the possibility subjects’ performance is based on generalization rather than a memory of a specific one-time event.

Pragmatically, the single trial criterion is unrealistic. Indeed, the apes cannot be verbally informed about the events that are about to unfold and it is difficult to see how they could possibly anticipate the fact they are about to be presented with a novel test. With respect to associative learning, one could argue that, at present, the data do not accord with this account. Indeed, the marked individual variation displayed by great apes is not consistent with an associative account, which one would expect to result in much more uniform performance. Further, an associative account would predict a decrease in performance across trials rather than the increase Suddendorf and Corballis (2010) suggest. As Osvath (2010) has pointed out, if associative learning was at play, the value of selecting the tool would quickly diminish due to the fact it cannot immediately be used to attain the reward it has been associated with. Finally, in one of the few studies to test monkeys, Dekleva et al. (2012) found that not a single monkey on a single trial, of the 17 test trials each were given, was able to pass the spoon test (**Table 1**). Again, given monkeys are quite adept at associative learning, an associative account would predict that monkeys would perform at a comparable level to great apes. Of course, additional studies will need to be conducted to ensure the failure of monkeys is due to the absence of mental time travel rather than a contextual variable (Bitterman, 1964). The failure of monkeys on the spoon test, however, is consistent with research looking at the ability of monkeys to plan over shorter time scales (Beran et al., 2004; Scarf and Colombo, 2009; Scarf et al., 2011a, b, 2014).

## CONCLUSION

In summary, if we apply the same criterion that has been used in developmental studies of mental time travel to studies conducted with great apes, it seems we must reject Tulving’s (2005) hypothesis that mental time travel is uniquely human.

## Conflict of Interest Statement

The authors declare that the research was conducted in the absence of any commercial or financial relationships that could be construed as a potential conflict of interest.

## Footnotes

1 The present review focuses on comparing the performance of children and non-human animals on comparable spoon test paradigms. Due to this we have not included the innovative series of studies conducted by Correia et al. (2007) and Raby et al. (2007) with scrub-jays. Future developmental studies may look to adopt a version of Clayton and colleagues’ paradigm to see if children’s performance mirrors that of the scrub-jays.

2 This approach makes the calculation of chance somewhat difficult due to the fact there is no clear chance value for transporting the tool. However, given that all of the studies we discuss coded tool transport as a binary outcome (i.e., either successful or unsuccessful), we set the chance value for tool transport at 0.5. To calculate an overall level of chance we then multiplied this value by the probability of selecting the correct tool by chance, which is simply a function of the number of target and non-target tools subjects had to choose from. The performance of each subject was assessed using a binomial probability test (see **Table 2**).
